# Tapping into alcohol use during COVID: Drinking correlates among bartenders and servers

**DOI:** 10.1371/journal.pone.0300932

**Published:** 2024-04-16

**Authors:** Rupa Jose, Weixi Wang, Garrick Sherman, Richard N. Rosenthal, H. Andrew Schwartz, Lyle H. Ungar, James R. McKay

**Affiliations:** 1 Positive Psychology Center, University of Pennsylvania, Philadelphia, Pennsylvania, United States of America; 2 Department of Computer Science, Stony Brook University, Stony Brook, New York, United States of America; 3 Department of Psychiatry, Stony Brook University, Stony Brook, New York, United States of America; 4 Department of Computer and Information Science, University of Pennsylvania, Philadelphia, Pennsylvania, United States of America; 5 Philadelphia Crescenz Veterans Affairs Medical Center, Philadelphia, Pennsylvania, United States of America; 6 Department of Psychiatry, University of Pennsylvania, Philadelphia, Pennsylvania, United States of America; Satyawati College (Eve.), University of Delhi, INDIA

## Abstract

The COVID pandemic placed a spotlight on alcohol use and the hardships of working within the food and beverage industry, with millions left jobless. Following previous studies that have found elevated rates of alcohol problems among bartenders and servers, here we studied the alcohol use of bartenders and servers who were employed during COVID. From February 12-June 16, 2021, in the midst of the U.S. COVID national emergency declaration, survey data from 1,010 employed bartender and servers were analyzed to quantify rates of excessive or hazardous drinking along with regression predictors of alcohol use as assessed by the 10-item Alcohol Use Disorders Identification Test (AUDIT). Findings indicate that more than 2 out of 5 (44%) people surveyed reported moderate or high rates of alcohol problem severity (i.e., AUDIT scores of 8 or higher)–a rate 4 to 6 times that of the heavy alcohol use rate reported pre- or mid-pandemic by adults within and outside the industry. Person-level factors (gender, substance use, mood) along with the drinking habits of one’s core social group were significantly associated with alcohol use. Bartenders and servers reported surprisingly high rates of alcohol problem severity and experienced risk factors for hazardous drinking at multiple ecological levels. Being a highly vulnerable and understudied population, more studies on bartenders and servers are needed to assess and manage the true toll of alcohol consumption for industry employees.

## Introduction

Excessive or hazardous drinking can impact all facets of one’s life by compromising work performance, relationships, and health [1]. In the U.S., the COVID-19 pandemic served as a catalyst for alcohol consumption, leading the average adult to drink more monthly in the early months of the pandemic [2]. The effect of the pandemic on alcohol use varied depending on study timing, population, and alcohol measures. Some studies indicated that alcohol use remained stable for certain sub-populations (e.g., for women) [3] or decreased, but most U.S. population studies suggested that the pandemic was linked to an overall increase in alcohol consumption [4]. Despite being a historically vulnerable industry for alcohol use pre-pandemic that was directly impacted by pandemic policies, little is known about the drinking behaviors of bartenders and servers during COVID. Understanding the drinking behaviors of bartenders and servers, along with the social-affective correlates of heavy drinking, supports pandemic recovery and helps prepare frontline workers in the event of future global health emergencies (“frontline”, a restaurant industry trade term, refers to those who engage directly with customers).

Occupational studies on alcohol use consistently find that bartenders and servers have among the highest rates of heavy alcohol use [5, 6] and alcohol-related deaths (i.e., death by liver cirrhosis [7]) when compared to other professionals. A variety of social, structural, and situational factors are believed to drive drinking vulnerability for bartenders and servers. For example, bartenders and servers are often called on to manage the emotional burdens and needs of patrons [8–10] or, conversely, forced to contend with violence, harassment, and bullying from patrons, peers, or supervisors [11, 12]. Shift-work, high job stress, low job autonomy, being embedded in a “drinking culture”, and having access to alcohol in the workplace are all thought to increase problem drinking among this population [1, 13, 14].

Two known drinking triggers were exacerbated with the pandemic: job loss [15] and employment insecurity. In 2018, food service, eating, and drinking establishments employed around 13 million Americans or 6.4% of all those employed [16]. By 2020, over 110,000 food and beverage establishments closed, resulting in the removal of 2.5 million jobs [17]. For those who managed to stay employed, the loss of tips/wages, potential virus exposure, and increased financial pressure may have turned some to drink–with a recent study finding employed restaurant workers drank significantly more than those furloughed during COVID [18]. Here we studied the risk factors associated with excessive or hazardous alcohol consumption among currently employed frontline bartenders and servers.

Decades of research on risk factors indicates that stress increases one’s motivation to drink alcohol along with the frequency of heavy drinking episodes [19, 20]. Chronic excessive alcohol exposure may itself act as a stressor, reinforcing further alcohol use [21]. Positive and negative affect can also influence alcohol consumption [22]. Young adults, for example, often attribute their drinking to coping with negative affect, and days with elevated positive or negative affect are associated with increased alcohol consumption [23–25]. Studies on clinical samples receiving treatment for alcohol problems find that depression and/or anxiety are associated with heavy alcohol use upon program entry and alcohol relapse [26–28]. The presence of social network members may likewise confer risk or protection depending on the alcohol behaviors of network ties [29, 30]. For adults, being connected to heavy drinkers can increase alcohol consumption by 70%, while surrounding oneself with abstainers can decrease consumption by half [29].

The present study examined alcohol use in bartenders and servers during the COVID pandemic. Drawing on prior literature, we analyzed the association of alcohol use with multiple measures of stress (i.e., perceived stress, stressful life events, covid stress), positive and negative affect, depression and anxiety, other substance use (tobacco, drug use, etc.), and the drinking behaviors of important social network members, to understand the individual/relational factors correlated with heavy alcohol use. Food and drinking establishments consistently account for the largest share of total profits garnered from the domestic agriculture, food, and related industries (i.e., 1.055 trillion from 2010 to 2020 [31]). Being able to better identify and address risks or deterrents to excessive drinking for bartenders and servers thus has the potential to improve *millions of lives* along with the U.S. economy.

## Materials and methods

### Participants

To be eligible for the current study, participants needed to be at least 18 years old, based in the US, and identify as a “bartender, server, or other front-of-house restaurant worker.” We partnered with several organizations and Facebook groups willing to share our study details with their listservs (i.e., the U.S. Bartenders Guild, National Bartenders Association, Texas Restaurant Association, University of Houston’s College of Hotel and Restaurant Management, Nashville Servers/Bartenders, and Vegas Bartenders and Servers), to recruit participants into our online Qualtrics survey administered from February 12^th^ to June 16^th^, 2021.

At the time of survey assessment, the U.S. was under the COVID national emergency declaration (issued January 31^st^, 2020 through May 11^th^, 2023). The COVID-19 pandemic was still considered a global public health emergency, though state issued stay-at-home orders were mostly no longer active. Bars and restaurants during this time had reopened but states varied in their restrictions for such establishments (capacity limits, requiring masks, etc.) [32].

Raw data from Qualtrics indicated 10,346 site “visitors” during the recruitment period. Removing individuals who failed attention checks, did not provide sufficient survey details (i.e., partial completers), spam entries, duplicates (identified using entered pay/contact information; retaining only the earliest of multiple responses), and other invalid entries (e.g., test entries) resulted in data from 1,199 participants who completed their survey from February 16^th^ to June 15^th^, 2021. To align with prior literature, additional filtering was done such that only those whose current job title included key terms referencing bartenders or servers (i.e., “bar”, “beer”, “cocktail”, “beverage”, “sommelier”, “mixologist”, “bottle”, “server”, “waitress”, and “bayhost”) were included into our analytical sample. Individuals who currently were unemployed or not working (e.g., those furloughed or laid off; *n* = 16) and those who concurrently or exclusively worked in management positions (e.g., managers, directors, or owners; *n* = 139) were excluded. This decision was made to better capture the experiences and hardships specific to those whose job duties involved working the bar or serving customers. This yielded a total of 1,010 participants; most of whom were in the bar industry exclusively (*n* = 644). A total of 154 individuals had jobs with both bar and server titles.

Our study was reviewed and approved by the Institutional Review Board at the University of Pennsylvania. All participants provided informed written consent and were compensated $15 for completing baseline data collection. Baseline data collection included survey responses along with the acquisition of social media posts, text messages, and identifying contact information (for payment). Sensitive language/social media data were not used in the present study.

### Measures

#### Personal demographics

Self-reported age, gender, race, Hispanic ethnicity, annual income (a 12-level categorical variable ranging from “less than $10,000” = 1 to “more than $150,000” = 12), and education (an 8-level categorical variable ranging from “less than high school” = 1 to “postgraduate or professional degree (MA, MS, PhD, MD, JD)” = 8) were reported.

#### Mental health

Measures of individual affect, perceived stress, depression, and anxiety were included. Affect was measured in the past week using the valid and reliable 10-item Positive and Negative Affect Schedule (PANAS [33]). Positive affect (sample α = 0.82) and negative affect (sample α = 0.80) scores ranged from 5 to 25. The higher the positive or negative affect score, the greater the positive or negative affect experienced. The 4-item Cohen’s Perceived Stress Scale [34] was used to assess perceived stress levels during the past month. Two positively phrased items (i.e., feeling things were “going their way” and being confident that they could handle their personal problems) were reversed-coded. All items were summed to create our perceived stress score measure (sample α = 0.79), ranging from 0 (no stress) to 16 (high stress). The 9-item Patient Health Questionnaire (PHQ-9 [35]; sample α = 0.90) and 7-item Generalized Anxiety Disorder (GAD-7 [36]; sample α = 0.93) were also used to assess depression and anxiety, respectively. All items ask participants to assess the frequency they felt bothered by different things or had unsettling feelings in the past two weeks. In both cases, a 4-point response scale ranging from “not at all” to “nearly every day” was used and items were summed to create depression (range: 0–27) and anxiety (range: 0–21) total scores. Higher score values were indicative of greater depression or anxiety.

#### Stressors and social factors

An adapted version of the 17-item Life Events Checklist for the DSM-5 (LEC-5 [37]) was used to capture one’s trauma history. Only removing one non-specific item (“severe human suffering”), participants were asked to endorse the other stressful life event items using the responses “happened to me”, “witnessed it”, or “doesn’t apply”. A count of the number of “happened to me” stressful life events (range: 0–16) were used in analyses. We also created a 6-item measure of COVID stressors keeping in mind the content of other COVID assessments [38]. Items focused on how COVID impacted individuals financially (e.g., income), socially (e.g., discrimination), or personally (e.g., death of loved ones). Across items, responses were coded such that higher values indicated greater COVID-related stress. All items were summed to create a COVID stressor severity score ranging from 0 (no COVID stress) to 10 (high COVID stress). Given the importance of the social group in encouraging or discouraging hazardous alcohol use, we included 4 items modified from the Important People Drug and Alcohol Interview (IPDA [39]). Participants were asked to “think of the 6 people (peers or other adults) who are most important to you in your life” and then indicate how many of those 6 people drank regularly, drank heavily, encouraged drinking or offered alcohol, and spent time in social activities weekly without drinking. The count of important people (range: 0–6) endorsing such behaviors served as the response.

#### Substance use

Participants reported their lifetime, recent (past year), and current substance use. Lifetime substance use history was measured via a single item that asked individuals whether they had been diagnosed by a doctor or received treatment for anxiety, depression, and/or a substance use problem. Those who indicated being treated exclusively for substance use problems (2.38%) as well as those with comorbid conditions (substance use and anxiety: 1.59%, substance use and depression: 1.49%, all three: 7.85%), were coded as 1 (mentioned percents based on non-missing data or *n* = 1,007). Recent or past year substance use was assessed using the first item from the NIDA-Modified ASSIST tool (NIDA Quick Screen [40]) which asked about usage frequency for tobacco products, prescription drugs for non-medical reasons (i.e., used not as prescribed), and illegal drugs. All responses were coded as either 0 (never) or 1 (once or twice, monthly, weekly, or daily/almost daily). Current alcohol specific substance use was captured using the valid and reliable 10-item Alcohol Use Disorders Identification Test (AUDIT; see [41]). AUDIT responses were summed to create our outcome AUDIT total scores ranging from 0–40 where higher values indicate a greater likelihood of alcohol problems or use disorders (sample α = 0.86).

AUDIT total scores can also be grouped into categories which reflect harmful alcohol consumption. Category designations are as follows: abstainers (scores = 0), low alcohol problems/use disorders” (scores = 1–7), moderate alcohol problems/use disorders (scores = 8–15), and high alcohol problems/use disorders (scores = 16 or more). For interpretation purposes, prevalence rates will rely on AUDIT category classifications.

### Analyses

Descriptive statistics are included as means, standard deviations, and percentages. An ordinary least squares (OLS) regression was estimated to understand the factors associated with alcohol problems/use disorders based on AUDIT-10 total scores. The tabled partial or final regressions included regression coefficient values, 95% confidence intervals (CI), significant *p*-value symbols (i.e., *p < 0.05, **p < 0.01, and ***p < 0.001), and measures of model fit (*F* statistic and *R*-squared values). Regressions were estimated on the full sample along with male/female subsamples. Final model standardized coefficients or Beta values (β) were also plotted on the full sample to show relative effect sizes differences across statistically significant predictors.

## Results

Descriptive statistics are presented in Table 1. As shown, our sample of bartenders and servers primarily included middle-aged, White women who had graduated high school and were earning under $50,000 a year. On average, participants endorsed greater positive affect compared to negative affect and only “mild” (i.e., PHQ-9/GAD-7 scores of 5–9) depression (*M* = 7.69; *SD* = 6.05) or anxiety (*M* = 6.68; *SD* = 5.61). Lifetime trauma was limited (*M* = 4.40, *SD* = 2.71; max value = 16) however all but 2 participants reported experiencing COVID-related stressors (*M* = 5.90; *SD* = 1.57). In the past year, nearly half of our participants reported using tobacco, over a third reported using illegal drugs, and 22% indicated they had misused prescription drugs. Additionally, many of our participants (44.05%) scored in the “moderate” or “high” range for AUDIT alcohol problems or use disorders [42]. Despite this, just 13% of people said they had been diagnosed by a doctor or received treatment for a substance use problem. The study sample treatment rate (13%) is however lower than the national rate in the 2021 National Survey of Drug Use and Health report (i.e., 15.6% of those 12 years old or older, and 15.1% of those 26 years old or older, reported either meeting DSM-5 criteria for an illicit drug/alcohol use disorder or receiving treatment at a specialized facility)–although this discrepancy might be because of differences in ages, item phrasing, and/or social desirability pressures [43]. Items inquiring about the behaviors within one’s intimate social circle (i.e., 6 important people) further showed that drinking was common practice (2–3 important people on average drank regularly, heavily, or encouraged/offered drinks) even though participants also had some people (2–3 important people on average) to spend time with on a weekly basis doing activities not centered on alcohol.

**Table 1:** Descriptive statistics (N = 1,010).

	*M*	*SD*	%
Demographics			
Age (years) ^a^	37.06	8.28	
Male ^b^			23.47
White race ^c^			79.41
Hispanic			15.35
Income			
Less than $30,000			27.82
$30,000 - $49,999			37.43
$50,000 - $69,999			19.31
$70,000 or more			15.45
Education			
Less than high school			0.59
Some high school (no degree)			1.78
High school graduate (diploma or GED)			14.16
Technical or vocational school			4.85
Some college or university (no degree)			33.86
Two-year Associates degree			12.67
Four-year Bachelors degree (BS, BA, AB)			27.13
Postgraduate or professional degree (MA, MS, PhD, MD, JD)			4.95
Affect and Mental Health			
Positive affect	16.52	4.30	
Negative affect	9.72	3.97	
Perceived Stress	6.53	3.35	
Depression ^d^	7.69	6.05	
Anxiety ^e^^,^^f^	6.68	5.61	
Stressful Life Events or COVID Stressors			
Count of Stressful Life Events ^e^	4.40	2.71	
Count of COVID stressors	5.90	1.57	
Support or Pressure to Drink from Important People (IP)			
Count of IP who drink regularly	3.09	1.80	
Count of IP who drink heavily (5+ drinks in single sitting)	2.00	1.61	
Count of IP who encourage/offer drinks	1.88	1.80	
Count of IP who spend time in non-drinking activities ^a^	2.58	1.90	
Substance Use (SU)			
SU history—lifetime ^g^			13.31
Tobacco use—past year			49.70
Prescription drug use—past year			22.38
Illegal drug use—past year			34.65
Alcohol Problems or Use Disorders^h^			
AUDIT total score	8.06	6.93	
AUDIT categories			
Abstainer (0)			9.60
Low alcohol problems/use disorders (1–7)			46.34
Moderate alcohol problems/use disorders (8–15)			29.50
High alcohol problems/use disorders (16+)			14.55

^a^ Two participants had missing data so excluded

^b^ Female: 75.54%, Other: 0.30%, Prefer not to say: 0.69%

^c^ Black or African American: 5.84%, Asian: 2.48%, American Indian or Alaskan Native: 1.29%, Native Hawaiian or other Pacific Islander: 0.40%, mixed race: 5.54%, other race or prefer not to say: 5.05%)

^d^ Minimal depression (0–4): 36.73%, mild depression (5–9): 29.31%, moderate depression (10–14): 19.41%, moderately severe depression (15–19): 9.11%, severe depression (> = 20): 5.45%

^e^ One participant missing all GAD-7 or LEC-5 items excluded

^f^ Minimal anxiety (0–4): 41.88%, mild anxiety (5–9): 31.19%, moderate anxiety (10–14): 15.25%, severe anxiety (> = 15): 11.58%

^g^ Percent based on non-missing data (*n* = 1,007)

^h^ Alcohol prevalence by gender: Male AUDIT total scores (*M* = 9.56, *SD* = 7.60), AUDIT categories (abstainer = 8.02%, low alcohol problems/use disorders = 41.35%, moderate alcohol problems/use disorders = 29.96%, high alcohol problems/use disorders = 20.68%); Female AUDIT total scores (*M* = 7.64, *SD* = 6.66), AUDIT categories (abstainer = 9.96%, low alcohol problems/use disorders = 47.71%, moderate alcohol problems/use disorders = 29.49%, high alcohol problems/use disorders = 12.84%)

*Note*. Table statistics shown as percentages (%), mean (*M*), and/or standard deviation (*SD*) values.

Demographic, affect, mental health, stressful life events, COVID stressors, social, and substance use variables were included in a linear regression model to understand which variables were associated with alcohol problems or use disorders among bartenders or servers. As shown in Table 2 (final model), those that identified as male (*b* = 1.59; *SE* = 0.44) or who have used substances in the past year (tobacco: *b* = 0.85; *SE* = 0.38; illegal drugs: *b* = 2.22; *SE* = 0.43) reported higher AUDIT scores than those who identified as female or reported no past year substance use, respectively. Higher recent negative affect (*b* = 0.16; *SE* = 0.07) or depression (*b* = 0.16; *SE* = 0.06) scores, and having close ties to drinkers (*b* = 0.31; *SE* = 0.15), especially those who drink heavily (*b* = 0.60; *SE* = 0.17) or tend to pressure one to drink (*b* = 0.75; *SE* = 0.14), were associated with higher AUDIT scores or indicative of greater alcohol problems or use disorders. In contrast, higher positive affect (*b* = -0.13; *SE* = 0.05) and having more people to socialize with in a non-drinking context (*b* = -0.32; *SE* = 0.10) was associated with lower AUDIT scores.

**Table 2:** Predicting drinking problems or use disorders in servers and bartenders (N = 1,001).

	Partial Models				Final Model
	Model 1.A	Model 1.B	Model 1.C	Model 1.D	Model 1.E
	*b* (95% *CI*)	*b* (95% *CI*)	*b* (95% *CI*)	*b* (95% *CI*)	*b* (95% *CI*)
Age (years)	-0.11 (-0.16, -0.06)***	-0.07 (-0.12, -0.03)**	-0.06 (-0.11, -0.01)*	-0.06 (-0.11, -0.01)*	-0.03 (-0.08, 0.01)
Male (= 1)	2.29 (1.28, 3.30)***	1.82 (0.86, 2.77)***	2.00 (1.07, 2.93)***	2.01 (1.08, 2.95)***	1.59 (0.72, 2.45)***
White (= 1)	1.20 (0.14, 2.27)*	0.80 (-0.20, 1.81)	0.70 (-0.28, 1.68)	0.69 (-0.29, 1.66)	0.28 (-0.63, 1.19)
Hispanic (= 1)	-0.76 (-1.96, 0.44)	-0.55 (-1.69, 0.58)	-0.50 (-1.60, 0.60)	-0.49 (-1.59, 0.61)	-0.08 (-1.10, 0.94)
Income	0.03 (-0.15, 0.21)	0.05 (-0.12, 0.22)	0.10 (-0.06, 0.27)	0.11 (-0.06, 0.27)	0.04 (-0.11, 0.19)
Education	0.38 (0.10, 0.65)**	0.35 (0.08, 0.61)*	0.29 (0.03, 0.55)*	0.29 (0.03, 0.54)*	0.16 (-0.07, 0.40)
History of substance use		0.21 (-0.99, 1.41)	-0.34 (-1.50, 0.83)	-0.36 (-1.54, 0.82)	0.33 (-0.76, 1.43)
Tobacco used past year		1.08 (0.25, 1.91)*	0.94 (0.13, 1.74)*	0.93 (0.12, 1.74)*	0.85 (0.11, 1.60)*
Prescription drugs used past year		1.97 (0.92, 3.01)***	1.27 (0.25, 2.29)*	1.29 (0.27, 2.31)*	0.89 (-0.05, 1.84)
Illegal drugs used past year		3.57 (2.65, 4.49)***	3.22 (2.32, 4.11)***	3.18 (2.28, 4.09)***	2.22 (1.37, 3.06)***
Positive affect			-0.12 (-0.22, -0.02)*	-0.12 (-0.22, -0.02)*	-0.13 (-0.22, -0.03)*
Negative affect			0.13 (-0.02, 0.29)	0.13 (-0.02, 0.29)	0.16 (0.02, 0.31)*
Perceived stress			-0.09 (-0.27, 0.08)	-0.09 (-0.27, 0.08)	-0.09 (-0.25, 0.08)
Depression			0.23 (0.11, 0.35)***	0.23 (0.11, 0.36)***	0.16 (0.04, 0.27)**
Anxiety			-0.01 (-0.15, 0.12)	-0.02 (-0.15, 0.12)	-0.03 (-0.16, 0.10)
Stressful life events				0.03 (-0.13, 0.19)	-0.07 (-0.22, 0.07)
COVID stressors				-0.08 (-0.34, 0.17)	-0.08 (-0.32, 0.15)
Count of 6 important people who drink regularly					0.31 (0.02, 0.60)*
Count of 6 important people who drink heavily					0.60 (0.27, 0.94)***
Count of 6 important people who encourage/offer drinks					0.75 (0.48, 1.02)***
Count of 6 important people who spend time in non-drinking activities					-0.32 (-0.51, -0.13)**
Constant	8.59 (6.00, 11.18)***	5.44 (2.90, 7.99)***	4.89 (1.48, 8.29)**	5.29 (1.63, 8.95)**	4.23 (0.82, 7.64)*
Overall Model Fit					
*F* statistic (*df*_model_, *df*_residual_)	7.67 (6,994)***	18.29 (10, 990)***	18.21 (15, 985)***	16.07 (17, 983)***	23.82 (21, 979)***
*R*^2^	0.04	0.16	0.22	0.22	0.34
Adjusted *R*^2^	0.04	0.15	0.21	0.20	0.32

*Note*. The above linear regression table includes unstandardized coefficient values (*b*), 95% confidence interval (CI) values, and overall model fit statistics. Fit statistics included the *F* statistic with degrees of freedom (*df*) values presented in parenthesis and R-squared (*R*^2^) values.

*p<0.05.

**p<0.01.

***p<0.001.

Final model results and the results of the partial models (where predictor blocks were entered in a sequential manner) are similar barring a few exceptions. Prior to the final model, negative affect was not statistically significant, age was negatively associated with AUDIT scores, and education along with past prescription drug use were positively associated with AUDIT scores. Across the partial models and full model, substance use and social network predictors resulted in the largest gains in *R*-squared–accounting for around a 12% increase each time in the model’s variance explained or predictive utility.

**Fig 1:**
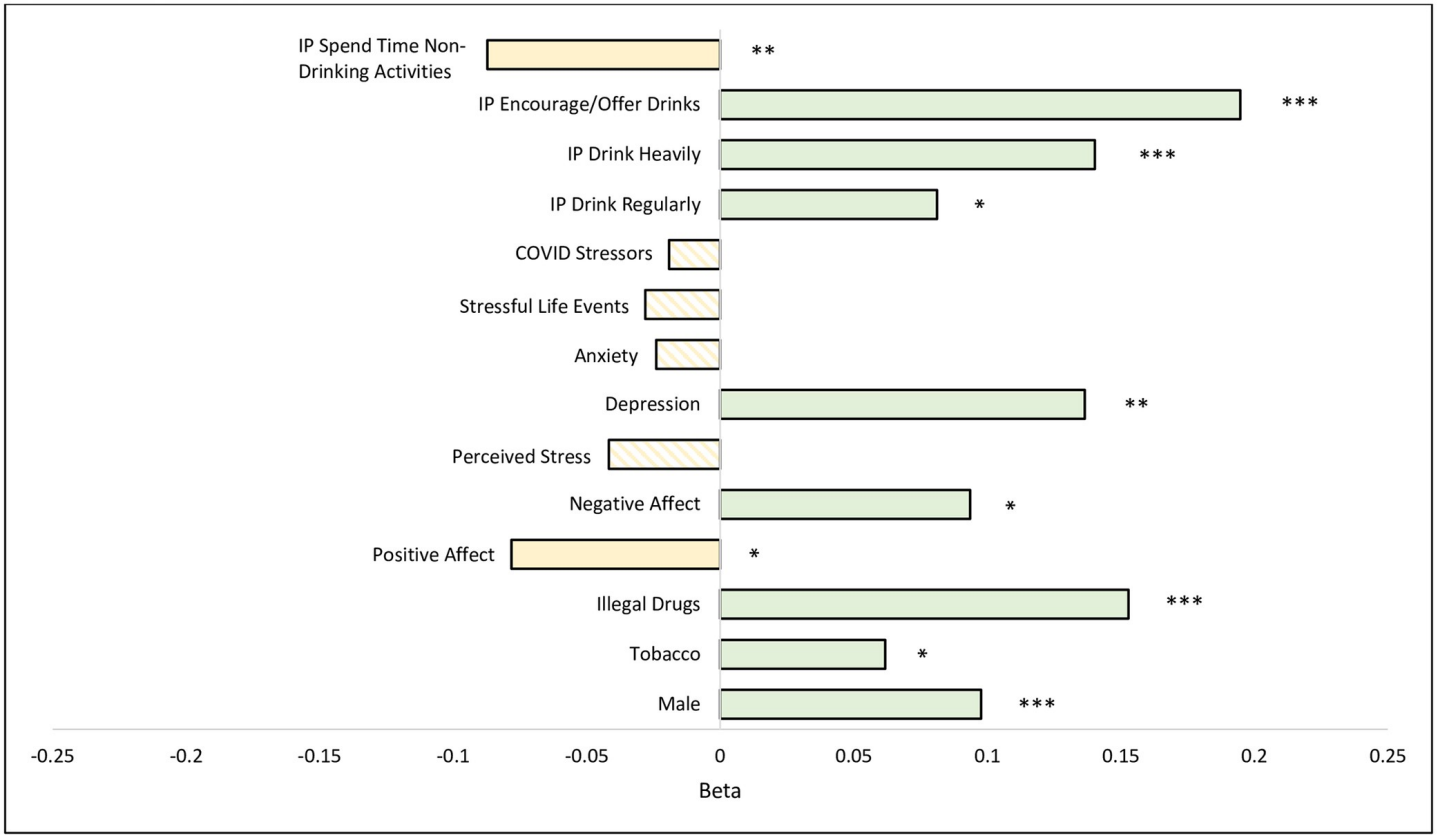
Bar graph of standardized predictors related to alcohol problems or use disorders (N = 1,001). The y-axis includes all statistically significant predictors of drinking problems or disorders in the final model: male gender, tobacco use past year, illegal drug use past year, positive affect, negative affect, depression, count of IP (important people) who drink regularly, count of IP who drink heavily, count of IP who encourage/offer drinks, and count of IP who spend time with one in non-drinking activities. It also includes a few key non-significant predictors measuring COVID stressors, stressful life events, anxiety, and perceived stress. The x-axis is standardized or Beta (β) values. *p<0.05. **p<0.01. ***p<0.001.

Plotted standardized coefficient final model values in Fig 1 show that social factors had the most robust associations with hazardous drinking. On the negative end, having people around you who encourage or offer drinks posed the greatest risk for problem drinking (β = 0.19); it was even worse than illegal drug use (β = 0.15) or being depressed (β = 0.14). On the positive end, having people around you to engage in activities that did not involve drinking (β = - 0.09) and being in a positive state of mind (β = - 0.08) appeared to be protective. No significant associations were found between AUDIT scores and COVID stress, stressful life events, anxiety, or perceived stress.

Results from our gender subsample models (see Table 3) suggested that while some of the risk factors for hazardous drinking were the same irrespective of gender (e.g., endorsing illegal drug use, having more core network members who offer/encourage drinking, and have few opportunities to socialize with close others without alcohol), not all risk factors are shared. For females, having close ties to more heavy drinkers (*b* = 0.68; *SE* = 0.19), endorsing a history of past prescription drug use (as opposed to no past use; *b* = 1.14; *SE* = 0.53), and higher depression scores (*b* = 0.14; *SE* = 0.06) were associated with higher AUDIT scores or problem drinking behavior. Additionally for females, higher positive affect scores (*b* = -0.14; *SE* = 0.05) were associated with lower AUDIT scores. For males, while having close ties with regular drinkers (*b* = 0.74; *SE* = 0.34) was associated with higher AUDIT scores, greater anxiety symptomatology (*b* = -0.35; *SE* = 0.17) was associated with lower AUDIT scores.

**Table 3:** Gender differences in alcohol problems or use disorders among servers and bartenders.

	Male (*N* = 236)	Female (*N* = 755)
	*b* (95% *CI*)	*b* (95% *CI*)
Age (years)	-0.04 (-0.15, 0.06)	-0.02 (-0.07, 0.03)
White (= 1)	-0.09 (-2.02, 1.84)	0.34 (-0.71, 1.40)
Hispanic (= 1)	0.24 (-1.82, 2.29)	-0.30 (-1.53, 0.92)
Income	-0.09 (-0.45, 0.26)	0.07 (-0.10, 0.25)
Education	-0.08 (-0.63, 0.46)	0.24 (-0.03, 0.51)
History of substance use	-1.57 (-3.95, 0.81)	1.09 (-0.17, 2.36)
Tobacco used past year	1.73 (-0.04, 3.50)	0.60 (-0.23, 1.43)
Prescription drugs used past year	0.34 (-1.99, 2.66)	1.14 (0.10, 2.17)*
Illegal drugs used past year	2.53 (0.43, 4.63)*	2.07 (1.13, 3.00)***
Positive affect	-0.04 (-0.27, 0.19)	-0.14 (-0.25, -0.03)**
Negative affect	0.28 (-0.05, 0.61)	0.13 (-0.03, 0.29)
Perceived stress	0.13 (-0.24, 0.51)	-0.14 (-0.33, 0.04)
Depression	0.30 (0.00, 0.61)	0.14 (0.02, 0.27)*
Anxiety	-0.35 (-0.69, -0.01)*	0.03 (-0.10, 0.17)
Stressful life events	-0.06 (-0.42, 0.30)	-0.06 (-0.22, 0.10)
COVID stressors	-0.20 (-0.74, 0.33)	-0.09 (-0.36, 0.18)
Count of 6 important people who drink regularly	0.74 (0.06, 1.41)*	0.24 (-0.09, 0.57)
Count of 6 important people who drink heavily	0.17 (-0.64, 0.98)	0.68 (0.31, 1.05)***
Count of 6 important people who encourage/offer drinks	0.89 (0.27, 1.52)**	0.70 (0.40, 1.01)***
Count of 6 important people who spend time in non-drinking activities	-0.62 (-1.12, -0.13)*	-0.23 (-0.43, -0.02)*
Constant	5.93 (-1.97, 13.83)	3.79 (-0.09, 7.68)
Overall Model Fit		
*F* statistic (*df*_model_, *df*_residual_)	6.60 (20, 215)***	17.80 (20, 734)***
*R*^2^	0.38	0.33
Adjusted *R*^2^	0.32	0.31

*Note*. The above linear regression table includes unstandardized coefficient values (*b*), 95% confidence interval (CI) values, and overall model fit statistics. Fit statistics included the *F* statistic with degrees of freedom (*df*) values presented in parenthesis and R-squared (*R*^2^) values. Alcohol problems and use disorders are measured by AUDIT-10 scores, in addition to males having on average higher AUDIT-10 scores (Male: *M* = 9.56, *SD* = 7.60; Female: *M* = 7.64, *SD* = 6.66) they also were more likely to endorse moderate or high alcohol problems (Male: 50.64%; Female: 42.33%) when compared to females.

*p<0.05.

**p<0.01.

***p<0.001

## Discussion

The present study found that almost half (44%) of employed bartenders and servers in the sample reported moderate to high rates of alcohol problem severity. Historic data on this population finds them at risk for alcohol problems such as alcohol abuse or dependence [5]. Consistent with this, our study reported rates for “high alcohol problems or use disorders” (14.55%) were notably higher whether we compared study rates to monthly pre-pandemic industry estimates of heavy alcohol use among employed adults (11.8% for adults in accommodations/food service industry [6]) or national rates of adult heavy alcohol use mid-pandemic (7% [44]). Even when looking at binge drinking, assessed with a single item from the NIDA Quick Screen [37] that asked how often one consumed alcohol (i.e., 5 or more drinks a day for men or 4 or more drinks a day for women) in the last year, we find that our sample of bartenders and servers binge drank (study rate: 84.65%) over 3.5 times more than the average American (24% [44]). Despite being a population at risk for an alcohol use disorder, only a fraction of bartenders and servers surveyed reported previously being diagnosed with or treated for a substance use disorder.

The strongest correlates of greater alcohol problem severity in the full sample were having important social network members who drank heavily or encouraged drinking, depression, negative affect, and using illegal drugs. The two factors significantly associated with lower alcohol problem severity were spending time in non-drinking activities with important social network members and positive mood. Surprisingly, our multiple measures of stress (perceived general stress, covid stress, and stressful life events) and anxiety were not significantly associated with problematic alcohol consumption.

The results linking social network drinking [29, 30], depression [26–28], and negative affect [22, 23, 25] to heavier drinking are consistent with findings from prior studies in clinical and non-clinical samples. The association between greater alcohol problem severity and illegal drug use is to be expected, given well-documented high rates of comorbidity between problematic alcohol and drug use as well as alcohol and drug use disorders [45]. Substance use comorbidity has been found across many populations and is not unique to bartenders and servers.

Population studies regularly suggest that COVID-related stress, perceived stress, life event stress, and anxiety can lead to alcohol use [4, 46, 47]; our null results however imply that the triggers for drinking among bartenders and servers during COVID have been different. Being employed in an industry where alcohol is often readily available, coupled with the decline in social and recreational activities [48] due to business closures and public health protocols, may have mattered more to our participants (most of whom were bartenders) than stress or anxiety when considering harmful alcohol consumption.

Contextual cues–whether social or structural, warrant future attention. Studies on alcohol use within this population may thus benefit from including multiple measures which approximate the social or physical environments that bartenders and servers navigate daily. COVID is an evolving global phenomenon which has changed, and continues to change, what it means to work in the service industry. Our results as such should be replicated.

Gender differences exist nationally [49] and in our sample (e.g., 50.64% of males vs. 42.33% of females reported “moderate” or “high” alcohol problems) for hazardous alcohol consumption. Risk factors also appear to be different along affective and social dimensions by gender. Although depression and anxiety are frequently associated with harmful drinking [50], in our sample it was only depressed women or those reporting low positive affect that were at risk for poor alcohol outcomes. Moreover, counterintuitively, for men self-reported recent anxiety was associated with a decline in alcohol problems. The sobering effect of anxiety on male bartenders or servers might signal for this subgroup heavy drinking only flourishes in times where one can be care-free (analogous to a “college” like effect) [51]. Feelings of anxiety might have also resulted in male bartenders or severs engaging in more health-conscious behaviors during the pandemic, including reduced drinking. Social network differences in risk also emerged with men being vulnerable for alcohol problems when around close *regular* drinkers and women being vulnerable for alcohol problems when around close *heavy* drinkers. This reinforces the idea that both network composition and degree of drinking play a role in drinking vulnerability for men and women [29]. Women who had abused prescription drugs in the past year reported higher AUDIT scores. Women and daily drinkers are more likely to abuse or misuse prescription drugs [52]. As women tend to use prescription drugs to cope with interpersonal trauma (e.g., sexual victimization) [53] or conflict [54], past prescription drug could be seen as a distal proxy for poor mental health.

Longitudinal research is needed to better understand how our established risk factors and alcohol consumption vary over time for bartenders, servers, and other service industry professionals. The correlational design of the current study is a limitation. In the absence of pre-pandemic alcohol use data, we were prevented from knowing the impact of pandemic on alcohol use and whether the alcohol use rates reported reflected participants’ status quo or a change (e.g., an increase in drinking). Still our recruitment of a large nationwide sample of currently employed bartenders or servers and the use of well-validated measures remains an important strength.

Recruiting participants from the food and beverage industry during the pandemic was challenging and resulted in a few additional limitations. For example, instead of recruiting in-person during national events or conventions regularly attended by bartenders and severs, we were forced to recruit using national or local industry listservs. This decision while appropriate could have biased our sample to include more socially connected/professionally integrated bartenders or servers. It also might have contributed to the sparse number of unemployed or furloughed individuals in the original data (*n* = 16; excluded in study analyses). Even though our focus was on those employed during the pandemic, our lack of a sizeable non-employed sample perhaps by design (as such individuals might not be active members of professional listservs) meant we were unable to comment on how pandemic job loss or employment change was associated with industry drinking rates. Importantly, we did not require participants to endorse recent alcohol use or a history of alcohol use, thus there is no reason to believe the high drinking rates reported reflect the sampling protocol. Findings are specific to employed bartenders/servers during the COVID pandemic and cannot be generalized to other service industry workers or national emergencies without additional research.

### Conclusion

The COVID pandemic reminded us how essential service professionals are and how fragile their health can be at times. Here we find that many bartenders and servers had been consuming alcohol at dangerous levels during the COVID pandemic. A mix of personal and social attributes such as depressed mood, negative affect, illegal substance use, and having close ties with drinkers or those who encourage drinking, were associated with more alcohol consumption among bartenders and servers while positive affect, self-reported anxiety, and sober socialization were found to be protective. The food and beverage industry is a multimillion-person enterprise with a risky drinker rate twice that of the general public (14.55% vs. 7% [44]). Attention and funds as such need to be directed at understanding and combating hazardous alcohol use for service industry professionals as it is a serious, pervasive health problem for many bartenders and servers. Gender tailored approaches are advocated for when tackling hazardous alcohol use among industry employees as rates along with risk or protective factors differ for male and female bartenders and servers.
